# Impact of acute kidney injury on survival in patients with chronic hepatitis C: a retrospective cohort study

**DOI:** 10.1186/s12879-021-05991-2

**Published:** 2021-03-25

**Authors:** Hankyu Jeon, Jae Heon Kim, Sang Soo Lee, Hee Jin Kim, Ra Ri Cha, Hyun Chin Cho, Jae Min Lee, Chang Yoon Ha, Hyun Jin Kim, Tae Hyo Kim, Woon Tae Jung, Ok-Jae Lee

**Affiliations:** 1 Department of Internal Medicine, Gyeongsang National University School of Medicine and Gyeongsang National University Hospital, Jinju, Republic of Korea; 2 Department of Internal Medicine, Gyeongsang National University School of Medicine and Gyeongsang National University Changwon Hospital, 11, Samjeongja-ro, Seongsan-gu, Changwon-si, Gyeongnam 51472 Republic of Korea; 3 Institute of Health Sciences, Gyeongsang National University, Jinju, Republic of Korea

**Keywords:** Hepatitis C virus, Mortality, Incidence, Acute kidney injury

## Abstract

**Background:**

Acute kidney injury (AKI) is expected to occur commonly in patients with chronic hepatitis C. In addition, AKI may affect the survival of patients with chronic hepatitis C. However, few studies are available on this topic. We aimed to evaluate the incidence of AKI in patients with chronic hepatitis C and investigate the factors related to overall mortality.

**Methods:**

Between January 2005 and December 2018, 1252 patients with chronic hepatitis C virus (HCV) infection, defined as persistent HCV RNA for at least 6 months, were retrospectively enrolled at two centers. Of them, 1008, 123, and 121 patients had chronic hepatitis (CH), compensated cirrhosis (Com-LC), and decompensated cirrhosis (Decom-LC) or hepatocellular carcinoma (HCC) at entry, respectively. Factors associated with AKI and overall mortality were evaluated using the Cox proportional regression model. The Kaplan-Meier survival curves for the development of AKI and overall mortality were generated.

**Results:**

Over a mean follow-up period of 5.2 years, 285 patients developed AKI, with an incidence rate of 4.35 per 100 person-years. The incidence of AKI increased gradually with progression of chronic hepatitis C: CH (3.32 per 100 person-years), Com-LC (5.86 per 100 person-years), and Decom-LC or HCC (17.28 per 100 person-years). The patients without AKI showed better survival rates at 14 years than the patients with AKI (94.2% vs. 26.3%, *P* < 0.001). In multivariate Cox regression analysis, AKI (hazard ratio, 6.66; 95% confidence interval, 4.26–10.41) remained an independent risk factor for overall mortality.

**Conclusion:**

AKI is common in patients with chronic HCV infection and is associated with significant overall mortality. Therefore, clinicians should carefully monitor the occurrence of AKI, which is an important predictor of mortality in patients with chronic hepatitis C.

**Supplementary Information:**

The online version contains supplementary material available at 10.1186/s12879-021-05991-2.

## Background

Renal impairment occurs in approximately 20–50% of hospitalized patients with cirrhosis [1–4]. Acute kidney injury (AKI) is a frequent and life-threatening complication in patients with cirrhosis associated with significant mortality [3, 5–7]. In particular, 30-day mortality is 10-fold higher among patients with persistent AKI [8]. Although most studies evaluating AKI in patients with cirrhosis included only those who are hospitalized, two studies focused on AKI in outpatients with cirrhosis [7, 9]. Even in stable outpatients with cirrhosis, 30–50% of patients developed AKI.

The presence of hepatitis C virus (HCV) infection increases not only the risk of AKI development, but also of chronic kidney disease development and prognosis [10, 11]. AKI often occurs in the natural history of chronic hepatitis C and may be associated with poor prognosis. According to previous studies reported in patients with cirrhosis, AKI may have a significant effect on mortality in patients with chronic hepatitis C. A study focused on AKI in patients with chronic hepatitis C reported a prevalence of 13.5% (64/468) [12]. However, data evaluating the incidence of AKI according to disease progression in patients with chronic hepatitis C are limited. As hepatitis C infection progresses from chronic hepatitis (CH) to decompensated cirrhosis (Decom-LC) or hepatocellular carcinoma (HCC) with progression of liver disease, the incidence of AKI is expected to increase. Further research is warranted to determine whether AKI is an important factor in mortality in patients with chronic hepatitis C.

We established a retrospective, longitudinal HCV cohort study in Korea to investigate the incidence of AKI in patients with chronic hepatitis C. The aims of the study were to elucidate the incidence of AKI in patients with chronic hepatitis C according to the natural course of liver disease and to investigate the factors related to overall mortality.

## Methods

### Study population

Between January 2005 and December 2018, a retrospective cohort of the natural history of liver disease progression was established in 1743 consecutive patients. The patients had a detectable HCV RNA of chronic hepatitis C diagnosed at two centers. Patients with at least two or more creatinine measurements obtained more than 3 months apart were included. Of these patients, 491 met any of the following exclusion criteria: (1) a follow-up period of less than 6 months without mortality (*n* = 390); (2) prior kidney transplant or end-stage renal disease (*n* = 23); (3) < 18 years of age (*n* = 2); (4) seropositivity for hepatitis B virus surface antigen (*n* = 70); or (5) seropositivity for the human immunodeficiency virus (HIV) (*n* = 6). The remaining 1252 patients with chronic hepatitis C infection were finally included in the analysis. The laboratory test results and comorbidities including diabetes and hypertension were extracted from the electronic medical records. The patients’ medical histories were invesgated to identify age, gender, alcohol consumption, and antiviral therapy. This study was approved by the Institutional Review Boards of two centers, Gyeongsang National University Changwon Hospital and Gyeongsang National University Hospital.

### Follow-up and definition

Retrospective chart reviews were performed to determine the natural history of liver disease progression and AKI occurrence. The index date was the date of the first positive HCV RNA for this analysis. After enrollment, all patients underwent blood tests and imaging examinations for HCC surveillance every 3–6 months. Antiviral therapy using pegylated interferon alpha and ribavirin (before 2014) and direct-acting agents (since 2014) was administered to patients in whom it was indicated according to the clinical decisions of the treating physicians. In our study, sustained virologic response (SVR) was defined as a serum HCV RNA test below limit of detection performed at least 12 weeks after the end of treatment.

Liver cirrhosis was determined as (1) presence of clinical sign of portal hypertension manifested as ascites, varices, or hepatic encephalopathy or (2) compatible imaging findings accompanied by thrombocytopenia (< 100,000/μL). Hepatic decompensation was defined by the presence of hepatic encephalopathy, ascites, hepatorenal syndrome, or documented gastro-esophageal variceal bleeding. Chronic hepatitis C infection was classified as (1) CH, (2) compensated cirrhosis (Com-LC), and (3) Decom-LC or HCC according to liver disease progression.

### Definition of acute kidney injury

AKI was defined according to the International Club of Ascites (ICA)-AKI criteria for patients with cirrhosis and to the Kidney Disease Improving Global Outcomes criteria for patients with CH [13, 14]. It was defined as the increase in serum creatinine of ≥0.3 mg/dL or ≥ 50% over baseline. For this purpose, baseline serum creatinine was defined as the most recent stable value within 3 months prior to hospitalization. In patients without a serum creatinine value within the previous 3 months, the last stable creatinine value between 3 months and 1 year was used as the baseline [1, 3]. If outpatient baseline values were not available, the creatinine value at admission was used as baseline if it was normal.

The AKI stages for severity were classified as follows: [15] AKI stage 1, serum creatinine increase ≥0.3 mg/dL or increases 1.5–1.9 times baselines; AKI stage 2, serum creatinine increase 2.0–2.9 times baseline; and AKI stage 3, serum creatinine increase 3.0 times beseline or serum creatinine ≥4.0 mg/dL or initiation of renal replacement therapy. When multiple episodes of AKI occurred in one patient, we selected an episode of AKI with a peak creatinine for convenience of statistics.

Subjects with AKI were classified into 4 types according to the cause of kidney impairment: (1) pre-renal AKI, (2) hepatorenal syndrome (HRS)-AKI, (3) intrinsic-renal AKI, and (4) post-renal AKI. The diagnostic criteria of HRS-type AKI were defined using the ICA criteria as previously reported [13].

### Statistical analysis

Statistical analyses were performed using PASW software (version 18; SPSS Inc., Chicago, IL). The Mann-Whitney *U* test was performed to analyze quantitative variables. Fisher’s exact and Pearson’s chi-square tests were conducted to analyze qualitative data. The incidence rate of AKI per 100 person-years was calculated by dividing the number of newly developed AKI event cases by the person-years of follow-up. In our study, the time to event was calculated from the date of enrollment to the study to the date of AKI occurrence, death, or last observation or to December 31, 2019, in each diagnosis group at entry. Survival curves for the development of AKI and overall mortality were calculated using the Kaplan-Meier method and compared with log-rank test. Univariate and multivariate analyses were conducted using the Cox proportional regression model to identify potential factors associated with AKI and overall mortality. To assess the risk factors associated with AKI, variables including age, gender, presence of hypertension and diabetes, alcohol consumption, HCV genotype, diagnosis at entry (CH, Com-LC, and Decom-LC or HCC), extrahepatic malignancy, SVR, HCV RNA, albumin, bilirubin, platelet, INR, Child-Pugh score, and MELD score were used in Cox univariate regression. Moreover, to assess the risk factors associated with overall mortality, Cox univariate analysis was performed by adding the presence of AKI to the existing variables for AKI. The significant variables (*P* < 0.05) by univariate analysis were subjected to Cox multivariate analysis. The risk was expressed as hazard ratio (HR) and 95% confidence interval (CI). A two-sided *P* value of < 0.05 was considered statistically significant for all analyses.

## Results

### Patient characteristics

A total of 1252 patients with chronic hepatitis C were followed up for a mean of 5.2 years. Baseline characteristics are shown in Table 1. Median age was 58 years, and 54.7% of the patients were male. Prevalence of hypertension and diabetes at baseline were 25.6 and 22.8%, respectively. At entry, CH was diagnosed in 1008 patients (80.5%); Com-LC, 123 patients (9.8%); and Decom-LC or HCC, 121 patients (9.7%).

**Table 1:** Baseline characteristics according to acute kidney injury

	All patients (*n* = 1252)	AKI (*n* = 285)	No AKI (*n* = 967)
Age, year ^a^	58.0 (49.0–67.8)	65.0 (54.0–71.0)	57.0 (48.0–66.0)
Male sex^a^	685 (54.7%)	176 (61.8%)	509 (52.6%)
Hypertension ^a^	321 (25.6%)	112 (39.3%)	209 (21.6%)
Diabetes ^a^	286 (22.8%)	108 (37.9%)	178 (18.4%)
Alcohol > 40 g/day	244 (19.5%)	65 (22.8%)	179 (18.5%)
Diagnosis at entry ^a^			
CH	1008 (80.5%)	186 (18.5%)	822 (81.5%)
Com-LC	123 (9.8%)	34 (27.6%)	89 (72.4%)
Decom-LC or HCC	121 (9.7%)	65 (53.7%)	56 (46.3%)
Genotype			
1	545 (43.5%)	108 (37.9%)	437 (45.2%)
2	603 (48.2%)	150 (52.6%)	453 (46.8%)
3	101 (8.1%)	26 (9.1%)	75 (7.8%)
Others	3 (0.2%)	1 (0.4%)	2 (0.2%)
Extrahepatic malignancy ^a^	90 (7.2%)	35 (12.3%)	55 (5.7%)
SVR ^a^	606 (48.4%)	49 (17.2%)	557 (57.7%)
HCV RNA > 600,000 *IU/mL*	202 (16.1%)	42 (14.7%)	160 (16.5%)
Albumin, *g/dL* ^a^	4.1 (3.7–4.4)	3.7 (3.1–4.1)	4.2 (3.9–4.5)
Bilirubin, *mg/dL* ^a^	0.69 (0.50–1.00)	0.77 (0.50–1.25)	0.67 (0.50–0.94)
Platelet, *×10*^*9*^*/L* ^a^	183.0 (131.0–232.0)	151.0 (102.0–215.0)	190.0 (142.0–233.0)
PT-INR ^a^	1.03 (0.99–1.11)	1.09 (1.02–1.22)	1.01 (0.98–1.09)
Child-Pugh B or C at entry ^a^	112 (8.9%)	66 (58.9%)	46 (41.1%)
MELD score at entry ^a^	7.0 (6.0–8.0)	8.0 (7.0–10.0)	7.0 (6.0–8.0)
Follow-up period (year)	4.2 (2.1–7.9)	4.6 (2.1–8.0)	4.1 (2.0–7.8)

Data are presented as median (interquartile range) for continuous data and percentages for categorical data

*Abbreviations*: *CH* Chronic hepatitis, *Com-LC* Compensated cirrhosis, *Decom-LC* Decompensated cirrhosis, *HCC* Hepatocellular carcinoma, *SVR* Sustained virologic response, *HCV* Hepatitis C virus, *PT-INR* Prothrombin time-international normalized ratio, *MELD score* Model For End-Stage Liver Disease score

^a^ *p* < 0.05 AKI vs No AKI using the Mann-Whitney *U* test and Chi-squared test

### Characteristics of subjects with AKI

Of the 1252 patients, 285 (22.8%) developed AKI. Patients with AKI were older than those without AKI. The proportions of male sex, hypertension, diabetes, and extrahepatic malignancy were higher in patients with AKI than in those without AKI. The risk of AKI incidence gradually increased as chronic hepatitis progressed to advanced liver disease.

A total of 149 (52.3%), 80 (28.1%), and 56 (19.6%) of the 285 patients had initial AKI stage 1, stage 2, and stage 3, respectively. During hospitalization, 121 (42.5%) patients had a peak AKI stage 1, 76 (26.7%) patients had a peak AKI stage 2, and 88 (30.9%) patients had a peak AKI stage 3. A resolution of AKI occurred in 77.7, 53.9, and 31.8% of the patients with peak AKI stages 1, 2, and 3, respectively (Supp. Figure 1). Dialysis-requiring AKI was observed in 26 (2.1%) patients.

The causes of AKI were: (1) pre-renal AKI in 127 (44.6%) patients, (2) HRS-AKI in 44 (15.4%) patients, (3) intrinsic-renal AKI in 108 (37.9%) patients, and (4) post-renal AKI in 6 (2.1%) patients.

### Incidence of AKI

**Fig. 1:**
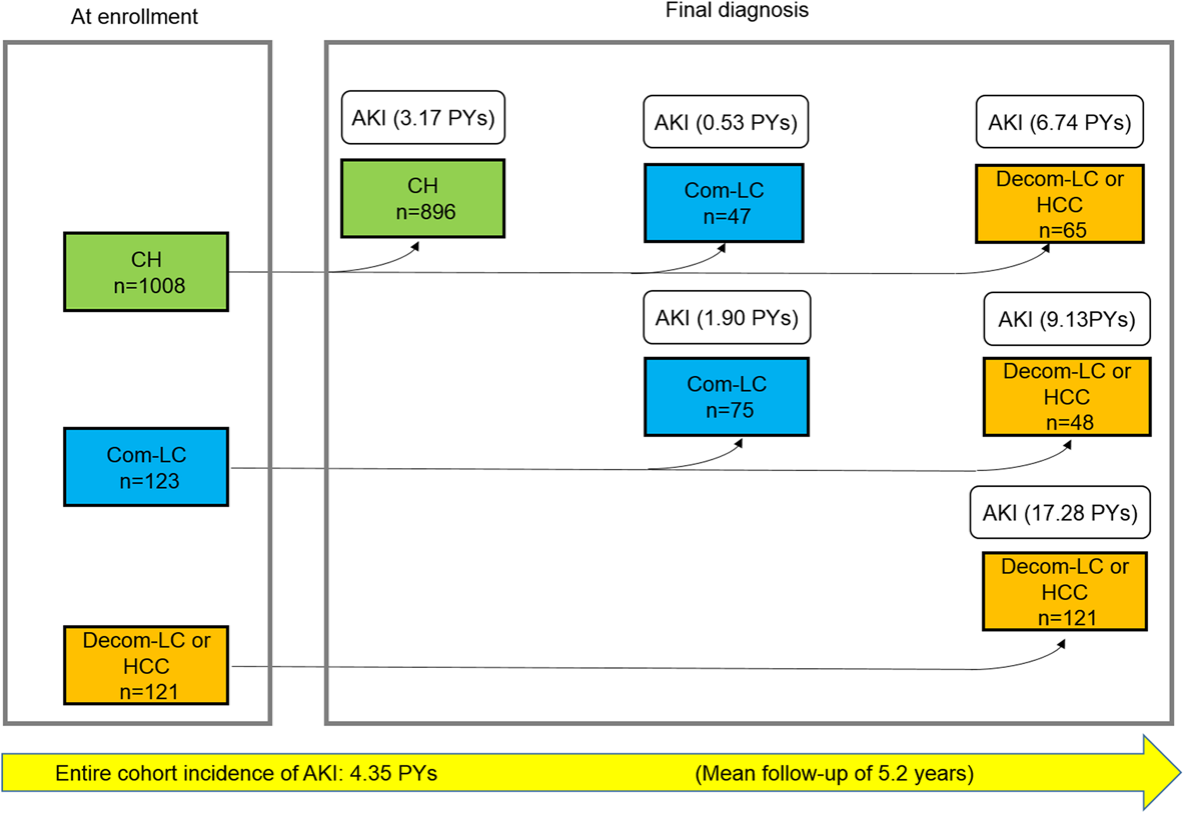
Incidence rate of AKI according to the natural history of chronic hepatitis C (*n* = 1252). The incidence of AKI is higher in patients with Decom-LC or HCC than in patients with CH and Com-LC. AKI incidence was calculated from the date of enrollment. CH, chronic hepatitis; Com-LC, compensated cirrhosis; Decom-LC, decompensated cirrhosis; HCC, hepatocellular carcinoma; PYs, per 100 person-years; AKI, acute kidney injury

**Fig. 2:**
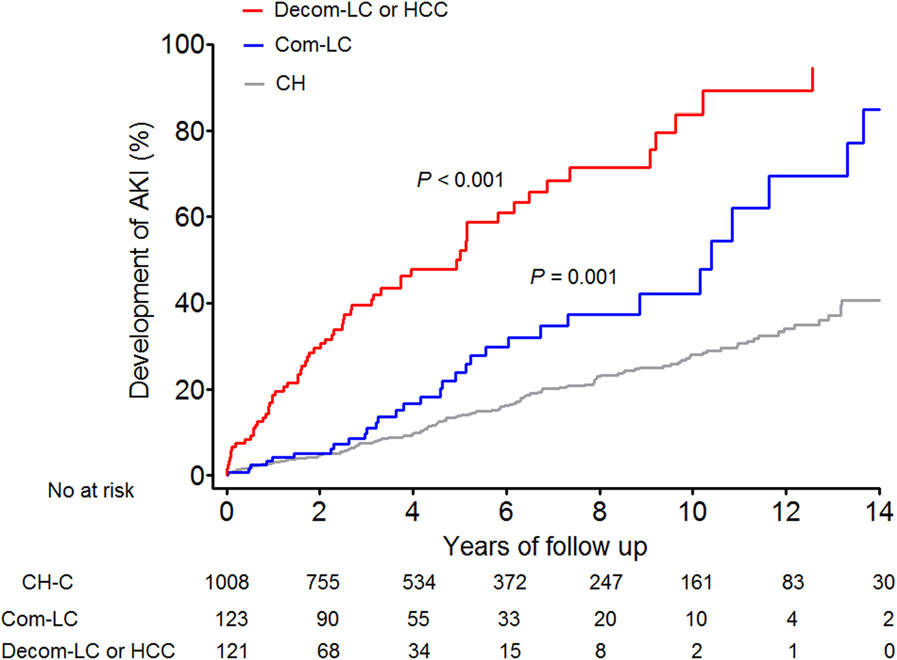
Cumulative incidence of AKI according to diagnosis at entry (*n* = 1252). Cumulative incidence of AKI is higher in patients with Com-LC and Decom-LC or HCC than in patients with CH. CH, chronic hepatitis; Com-LC, compensated cirrhosis; Decom-LC, decompensated cirrhosis; HCC, hepatocellular carcinoma; AKI, acute kidney injury

Over the follow-up of 6551.0 person-years, 285 of the 1252 patients developed new AKI, with an incidence rate of 4.35 per 100 person-years. The incidence rate of AKI would increase as liver disease progressed from CH to Com-LC and Decom-LC or HCC. Of the 1008 patients with CH at entry, 896 remained with CH, with an incidence rate of 3.17 per 100 person-years; 47 progressed to Com-LC, with an incidence rate of 0.53 per 100 person-years; and 65 progressed to Decom-LC or HCC, with an incidence rate of 6.74 per 100 person-years. Of the 123 patients with Com-LC at entry, 75 progressed to Com-LC, with an incidence rate of 1.90 per 100 person-years; and 48 progressed to Decom-LC or HCC, with an incidence rate of 9.13 per 100 person-years. The remaining 121 patients with Decom-LC or HCC at entry had an incidence rate of 17.28 per 100 person-years (Fig. 1). The overall incidence of AKI was higher in patients with Decom-LC or HCC (17.28 per 100 person-years, 95% CI = 13.09–21.49; *P* < 0.001) and with Com-LC (5.86 per 100 person-years, 95% CI = 3.88–7.81; *P* = 0.001) than in patients with CH (3.32 per 100 person-years, 95% CI = 2.87–3.83) (Fig. 2).

### Risk factors for AKI

The associated risk factors for the development of AKI in univariate analysis were male sex (HR, 1.32; *P* = 0.022), age (HR, 1.03 per year; *P* < 0.001), hypertension (HR, 1.58; *P* < 0.001), diabetes mellitus (HR, 1.97; *P* < 0.001), platelet count (HR, 0.99 per 10^9^/L; *P* < 0.001), Model For End-Stage Liver Disease (MELD) score at entry (HR, 1.15 per point; *P* < 0.001), Child-Pugh score at entry (HR, 1.72 per point; *P* < 0.001), SVR (HR, 0.21; *P* < 0.001), extrahepatic malignancy (HR, 1.73; *P* = 0.002), Com-LC at entry (HR, 1.83; *P* < 0.001), and Decom-LC or HCC at entry (HR, 5.49; *P* < 0.001). In the final multivariate analysis for the development of AKI, the risk factors included age (HR, 1.01 per year; *P* = 0.041), hypertension (HR, 1.49; *P* = 0.003), diabetes mellitus (HR, 1.31; *P* = 0.041), MELD score at entry (HR, 1.06 per point; *P* = 0.035), Child-Pugh score at entry (HR, 1.26 per point; *P* < 0.001), SVR (HR, 0.31; *P* < 0.001), extrahepatic malignancy (HR, 1.65; *P* = 0.009), and Decom-LC or HCC at entry (HR, 2.47; *P* < 0.001) (Table 2).

**Table 2:** Univariate and multivariate analyses showing significant predictive factors of acute kidney injury (*n* = 1252)

Variable	Univariate analysis		Multivariate analysis	
	*P*	HR (95% CI)	*P*	HR (95% CI)
Male	0.022	1.32 (1.04–1.68)	0.057	1.28 (0.99–1.64)
Age per year	< 0.001	1.03 (1.02–1.04)	**0.041**	1.01 (1.00–1.02)
Hypertension	< 0.001	1.58 (1.24–2.00)	**0.003**	1.49 (1.14–1.94)
Diabetes	< 0.001	1.97 (1.55–2.51)	**0.041**	1.31 (1.01–1.69)
Platelet per 10^9^/L	< 0.001	0.99 (0.99–1.00)	0.348	0.99 (0.99–1.01)
MELD score per point	< 0.001	1.15 (1.13–1.18)	**0.035**	1.06 (1.00–1.11)
Child-Pugh score per point	< 0.001	1.72 (1.59–1.86)	**< 0.001**	1.26 (1.09–1.46)
SVR	< 0.001	0.21 (0.16–0.29)	**< 0.001**	0.31 (0.23–0.43)
Extrahepatic malignancy	0.002	1.73 (1.22–2.47)	**0.009**	1.65 (1.13–2.41)
CH (diagnosis at entry)	Reference		Reference	
Com-LC	0.001	1.83 (1.26–2.65)	0.102	1.40 (0.94–2.09)
Decom-LC or HCC	< 0.001	5.49 (4.10–7.35)	**< 0.001**	2.47 (1.72–3.57)

*Abbreviations*: *HR* Hazard ratio, *CI* Confidence interval, *MELD score* Model for end-stage liver disease score, *SVR* Sustained virologic response, *Com-LC* Compensated cirrhosis, *Decom-LC* Decompensated cirrhosis, *HCC* Hepatocellular carcinoma

In our study, among the 400 patients treated with IFN, 10 (2.5%) developed AKI, and of the 347 patients treated with DAA, 7 (2.0%) developed AKI. Therefore, only a small number of patients who received antiviral therapy developed AKI. However, patients who achieved SVR through antiviral therapy had reduced risks of AKI. Of the 748 patients who started antiviral treatment after enrollment, 400 patients received pegylated interferon alpha and ribavirin treatment, with 291 patients (72.8%) achieving SVR, and 329 patients received direct-acting agents, with 302 patients (91.8%) achieving SVR (excluding 19 patients who continued antiviral treatment at the end of the study). In addition, 13 patients achieved SVR before study enrollment (Supp Table 1). The incidence of AKI was higher in patients who did not achieve SVR than those who achieved SVR (36.5% vs. 8.1%; *P* < 0.001). Among the 606 patients who achieved SVR, the incidence rate of AKI in patients with SVR before enrollment, patients treated with interferon and ribavirin, and patients treated with direct-acting agents was 7.7, 8.9, and 7.3%, respectively.

### Overall mortality

**Fig. 3:**
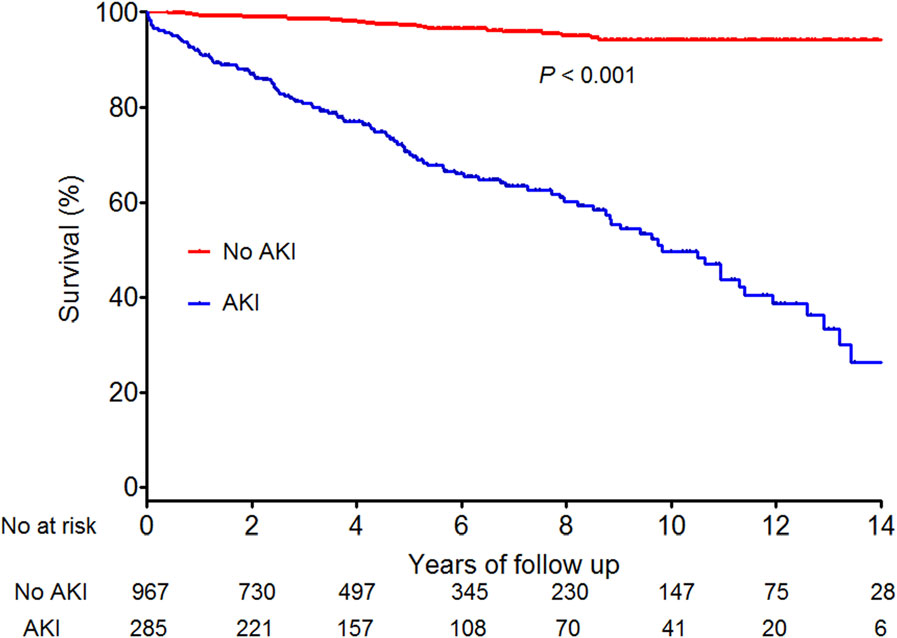
Survival outcomes in patients with chronic hepatitis C according to AKI (*n* = 1252). Patients without AKI had longer overall survival than patients with AKI. AKI, acute kidney injury

During the study period, 137 patients died (10.9%). The causes of mortality in patients with chronic hepatitis C were hepatic failure (*n* = 57), HCC (*n* = 25), extrahepatic malignancy (*n* = 23), sepsis (*n* = 23), and others (*n* = 9). The cumulative survival rate at 14 years was higher for patients without AKI (94.2%) than for patients with AKI (26.3%, *P* < 0.001) (Fig. 3).

The factors associated with overall mortality in univariate analysis were male sex (HR, 1.63; *P* = 0.007), age (HR, 1.03 per year; *P* < 0.001), platelet count (HR, 0.99 per 10^9^/L; *P* < 0.001), MELD score at entry (HR, 1.17 per point; *P* < 0.001), Child-Pugh score at entry (HR, 1.85 per point; *P* < 0.001), SVR (HR, 0.13; *P* < 0.001), extrahepatic malignancy (HR, 3.96; *P* < 0.001), Com-LC at entry (HR, 2.69; *P* < 0.001), Decom-LC or HCC at entry (HR, 12.13; *P* < 0.001), and AKI (HR, 13.60; *P* < 0.001). The factors associated with overall mortality in the final multivariate analysis included SVR (HR, 0.33; *P* < 0.001), extrahepatic malignancy (HR, 4.25; *P* < 0.001), Decom-LC or HCC at entry (HR, 5.63; *P* < 0.001), and AKI (HR, 6.66; *P* < 0.001) (Table 3).

**Table 3:** Univariate and multivariate analyses showing significant predictive factors of overall mortality (*n* = 1252)

Variable	Univariate analysis		Multivariate analysis	
	*P*	HR (95% CI)	*P*	HR (95% CI)
Male	0.007	1.63 (1.14–2.34)	0.097	1.38 (0.94–2.00)
Age per year	< 0.001	1.03 (1.02–1.05)	0.813	1.00 (0.99–1.02)
Platelet per 10^9^/L	< 0.001	0.99 (0.98–0.99)	0.590	0.99 (0.99–1.01)
MELD score per point	< 0.001	1.17 (1.14–1.20)	0.126	1.06 (0.98–1.14)
Child-Pugh score per point	< 0.001	1.85 (1.67–2.05)	0.436	1.08 (0.89–1.30)
SVR	< 0.001	0.13 (0.08–0.22)	**< 0.001**	0.33 (0.19–0.59)
Extrahepatic malignancy	< 0.001	3.96 (2.35–6.69)	**< 0.001**	4.25 (2.64–6.82)
CH (diagnosis at entry)	Reference		Reference	
Com-LC	< 0.001	2.69 (1.60–4.53)	0.108	1.60 (0.90–2.84)
Decom-LC or HCC	< 0.001	12.13 (8.27–17.79)	**< 0.001**	5.63 (3.39–9.37)
AKI	< 0.001	13.60 (8.92–20.73)	**< 0.001**	6.66 (4.26–10.41)

*Abbreviations*: *HR* Hazard ratio, *CI* Confidence interval, *MELD score* Model for end-stage liver disease score, *SVR* Sustained virologic response, *Com-LC* Compensated cirrhosis, *Decom-LC* Decompensated cirrhosis, *HCC* Hepatocellular carcinoma, *AKI* Acute kidney injury

Among the 285 patients with AKI, patients with HRS-AKI had the worst survival in comparison with the patients with prerenal-AKI (*P* < 0.001), intrinsic-renal AKI (*P* < 0.001), and postrenal-AKI (*P* < 0.001) (Supp. Figure 2). Moreover, patients with AKI stage 1 had longer overall survival than patients with AKI stage 2 (*P* < 0.001) and AKI stage 3 (*P* < 0.001) (Supp. Figure 3).

## Discussion

In our large retrospective longitudinal study with a long duration of follow-up involving patients with chronic HCV infection, we identified an AKI prevalence rate of 22.8%. The incidence rate of AKI in the entire cohort was 4.35 per 100 person-years (CH, 3.32 per 100 person-years; Com-LC, 5.86 per 100 person-years; and Decom-LC or HCC, 17.28 per 100 person-years). Patients without AKI had a better survival rate than patients with AKI. In multivariate analysis, AKI remained an independent risk factor for overall mortality (HR 6.66).

Few data are available on the incidence of AKI in patients with chronic hepatitis C [12]; previous studies focused on the HIV-HCV co-infection population [16, 17]. Garg et al. reported an AKI prevalence rate of 17%, with an incidence rate of 3.53 per 100 person-years in HCV-monoinfected subjects. Satapathy et al. reported that 63 patients experienced 124 episodes of AKI events among 468 patients with chronic HCV infection. The prevalence of AKI in our study was 22.8%, with 4.35 per 100 person-years, which is similar to those observed in previous studies. To the best of our knowledge, our study is the largest to report about the incidence rate of AKI in patients with chronic HCV infection.

We found an increasing incidence of AKI according to chronic HCV infection progression from CH to Com-LC, Decom-LC, or HCC (Figs. 1 and 2). This is consistent with the results that indicators of liver function, such as MELD score at entry, Child-Pugh score at entry, and Decom-LC or HCC at entry, are independent risk factors for the development of AKI (Table 2). Satapathy et al. reported that decompensated liver disease is a single viral-related factor for AKI in HCV-infected patients, and Garg et al. also reported that Decom-LC remains significantly associated with AKI in patients with HIV/HCV-coinfected patients in multivariate analysis [12, 17]. Interestingly, we showed that the achievement of SVR prevented the progression of AKI in patients with chronic HCV infection, which is a new result that has not been shown in previous studies (Table 2).

The most common causes of AKI were prerenal-AKI (44.6%), followed by intrinsic-AKI (37.9%), HRS-AKI (15.4%), and postrenal-AKI (2.1%). Mechanisms that explain the development of AKI in chronic hepatitis C include factors such as chronic impairment of kidney, poor liver function, ascites, low arterial pressure, low serum sodium, shock, high leukocyte, and use of antiviral agent [1, 11, 18]. We found that bacterial infection also contributes as a common precipitant of AKI in patients with chronic hepatitis C. However, this study was not designed to assess the mechanism of AKI development, and therefore we can only speculate on the mechanism of AKI development in the natural history of chronic hepatitis C.

We identified that AKI was also associated with a significant increase in the overall mortality. The peculiar result of our study was the approximately 5.6-fold increase (HR 6.66) in the risk of overall mortality in chronic HCV-infected patients with AKI compared with that in patients without AKI. A previous study by Nadkarni et al. using a nationally representative database reported that dialysis-requiring AKI is associated with a significant increase in in-hospital mortality among HCV-positive adults [19]. However, data evaluating the impact of AKI on mortality among HCV-positive individuals are limited. Our results suggest that clinicians should carefully monitor the development of AKI, which is an important predictor of mortality in patients with chronic hepatitis C.

This study has a few limitations. First, its retrospective design may limit the collection of factors related to AKI and mortality. Second, the irregular timing of laboratory assessment in each patient may cause missing AKI not found in this analysis (especially in patients in an outpatient setting). Despite these limitations, the strength of this study is that it is the largest study about AKI in patients with chronic HCV infection to date and that it showed the effect of AKI on mortality. Moreover, our study describes renal outcome in patients with chronic HCV infection in both outpatient and inpatient settings.

## Conclusion

Based on the findings of this large longitudinal cohort study, we found that the occurrence of AKI has a significant effect on mortality in the natural history of patients with chronic HCV infection. Our observation suggests that AKI events are common in the natural history of chronic hepatitis C, and that those with AKI should be monitored closely.

## Supplementary Information

**Additional file 1: Supp. Table 1.** Treatment outcome (*n*=1252).

**Additional file 2: Supp. Figure** 1**.** Course of acute kidney injury (*n*=285).

**Additional file 3: Supp. Figure 2.** Overall survival according to etiologies of acute kidney injury *(n*=285).

**Additional file 4: Supp. Figure 3.** Overall survival according to stage of acute kidney injury (*n* = 285).

## Data Availability

The datasets generated and/or analyzed during the current study are not publicly available due to ethical and confidentiality reasons but are available from the corresponding author on reasonable request under the Gyeongsang National University Changwon Hospital and Gyeongsang National University Hospital Ethics Committee’s approval. The data that support the findings of this study are available on request to the correspondence author. (Sang Soo Lee, Email:3939lee@naver.com).

## Abbreviations

AKI: Acute kidney injury
CH: Chronic hepatitis
CI: Confidence interval
Com-LC: Compensated cirrhosis
Decom-LC: Decompensated cirrhosis
HCV: Hepatitis C virus
HCC: Hepatocellular carcinoma
HIV: Human immunodeficiency virus
HR: Hazard ratio
HRS: Hepatorenal syndrome
ICA: International Club of Ascites
MELD: Model for end-stage liver disease
SVR: Sustained virologic response
