# Supplementary material for: Impact of acute kidney injury on survival in patients with chronic hepatitis C: a retrospective cohort study
Source: BMC Infect Dis. 2021 Mar 25;21:301. doi: 10.1186/s12879-021-05991-2 (PMC7993493; doi:10.1186/s12879-021-05991-2)
Supplement: Supplementary file 1 — Additional file 1: Supp. Table 1. Treatment outcome (n=1252). [file 12879_2021_5991_MOESM1_ESM.docx]

Supp. Table 1. Treatment outcome (n=1252)

| Variables | Total (n=1252) |
| --- | --- |
| Treatment regimen after enrollment ^a^ |  |
| No antiviral treatment | 504 (40.3%) |
| ^d^ Interferon and ribavirin | 400 (31.9%) |
| ^d^ Direct-acting agents | 348 (27.8%) |
| ^e^ SVR (n=1252) | 606 (45.4%) |
| IFN and ribavirin (n=400) | 291 (72.8%) |
| ^f^ Oral DAA (n=329) | 302 (91.8%) |
| SVR before enrollment | 13 |

^a^ last treatment regimen

^b^ excluding 19 patients who continued using the direct-acting agents treatment at the end of the study
