# Supplementary figures and images for: Impact of acute kidney injury on survival in patients with chronic hepatitis C: a retrospective cohort study
Source: BMC Infect Dis. 2021 Mar 25;21:301. doi: 10.1186/s12879-021-05991-2 (PMC7993493; doi:10.1186/s12879-021-05991-2)

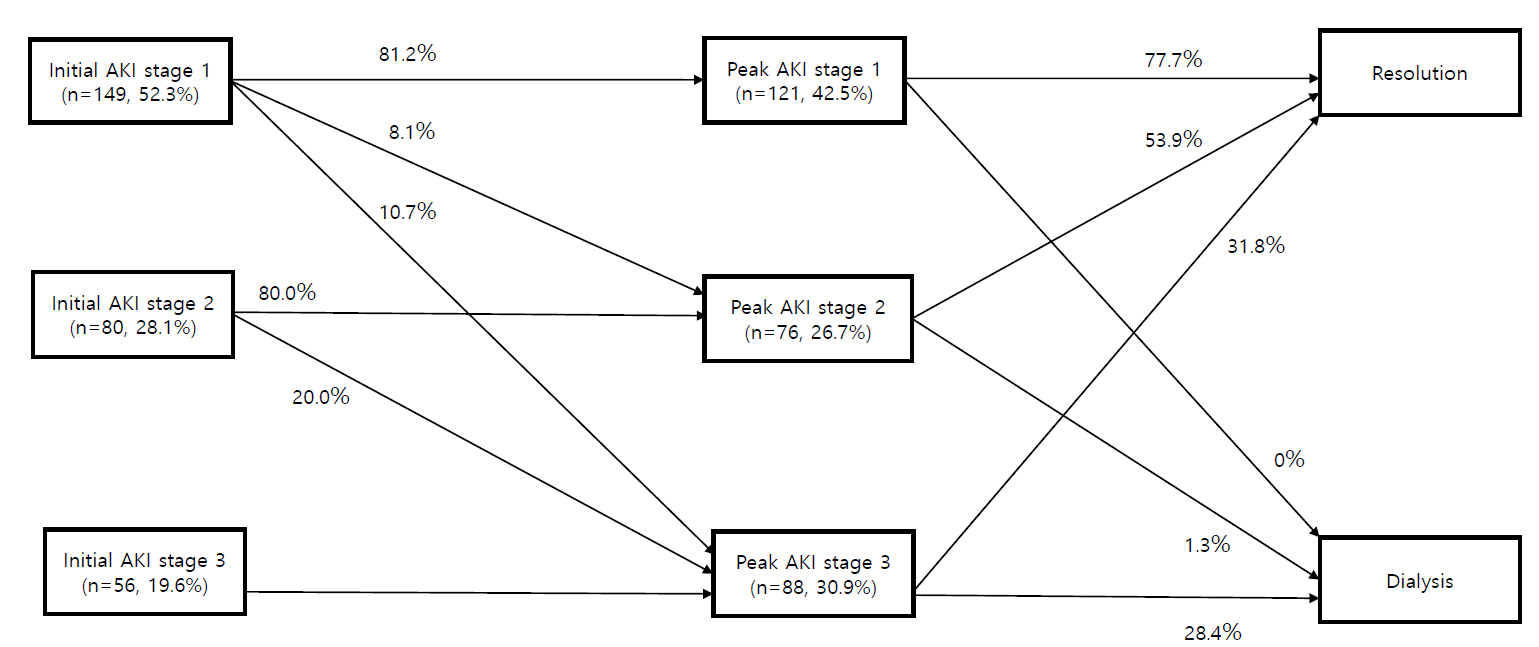

Supplement: Supplementary file 2 — Additional file 2: Supp. Figure 1. Course of acute kidney injury (n=285). [file 12879_2021_5991_MOESM2_ESM.tif]

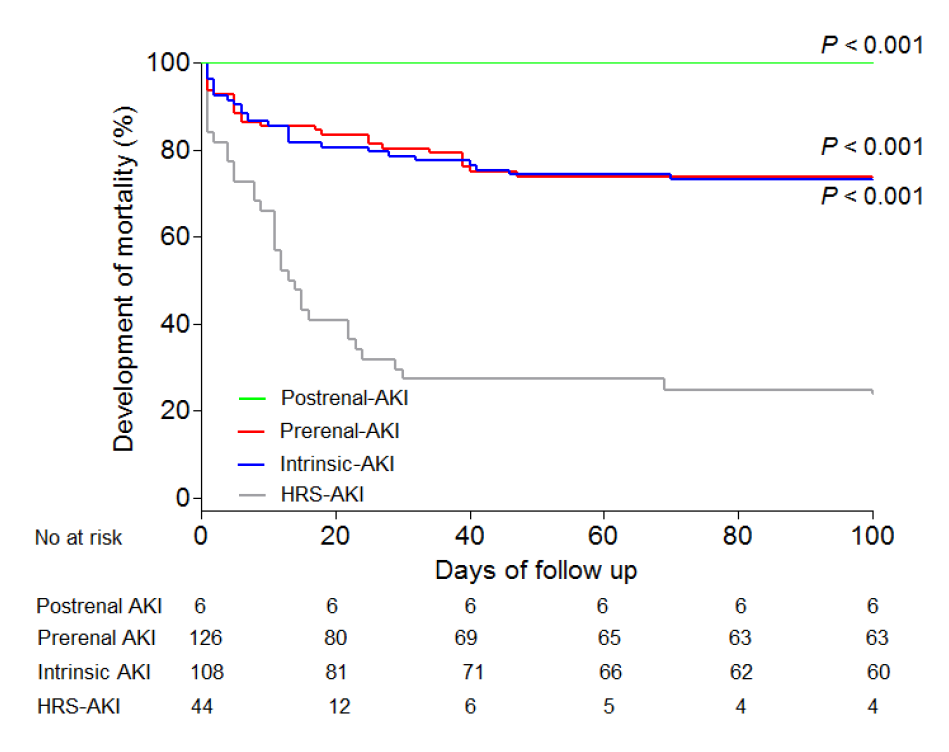

Supplement: Supplementary file 3 — Additional file 3: Supp. Figure 2. Overall survival according to etiologies of acute kidney injury (n=285). [file 12879_2021_5991_MOESM3_ESM.tif]

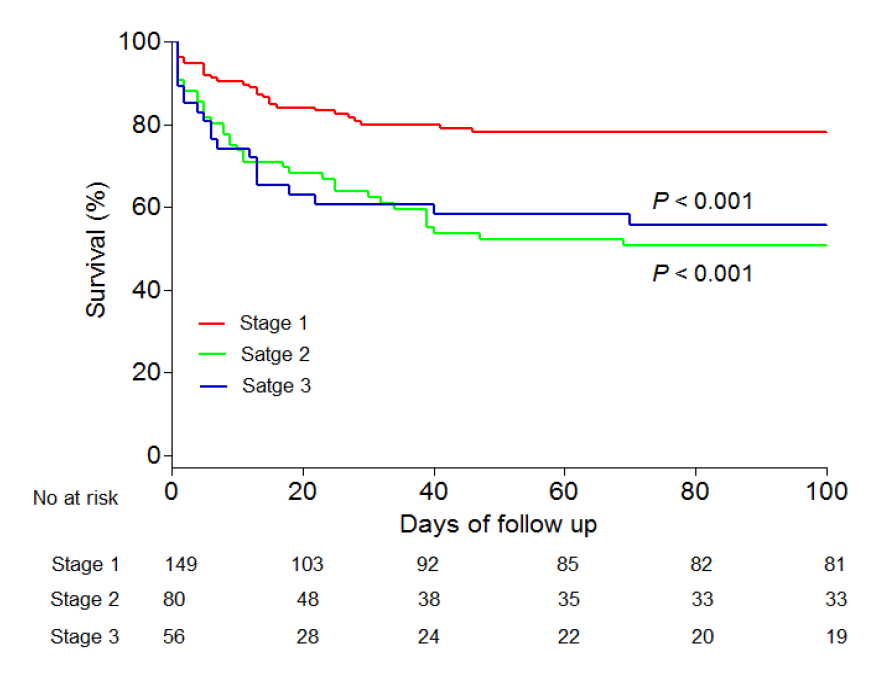

Supplement: Supplementary file 4 — Additional file 4: Supp. Figure 3. Overall survival according to stage of acute kidney injury (n = 285). [file 12879_2021_5991_MOESM4_ESM.tif]
